# Priming Adipose-Derived Mesenchymal Stem Cells with Hyaluronan Alters Growth Kinetics and Increases Attachment to Articular Cartilage

**DOI:** 10.1155/2016/9364213

**Published:** 2016-02-15

**Authors:** Peter Succar, Michael Medynskyj, Edmond J. Breen, Tony Batterham, Mark P. Molloy, Benjamin R. Herbert

**Affiliations:** ^1^Department of Chemistry & Biomolecular Sciences, Macquarie University, North Ryde, NSW 2109, Australia; ^2^Translational Regenerative Medicine Research Laboratory, Kolling Institute of Medical Research, Institute of Bone and Joint Research, University of Sydney at Royal North Shore Hospital, St Leonards, NSW 2065, Australia; ^3^Regeneus Ltd., 25 Bridge Street, Pymble, NSW 2073, Australia; ^4^Australian Proteome Analysis Facility, Macquarie University, North Ryde, NSW 2109, Australia; ^5^Quirindi Vet Clinic, 81 Pryor Street, Quirindi, NSW 2343, Australia

## Abstract

*Background*. Biological therapeutics such as adipose-derived mesenchymal stem cell (MSC) therapy are gaining acceptance for knee-osteoarthritis (OA) treatment. Reports of OA-patients show reductions in cartilage defects and regeneration of hyaline-like-cartilage with MSC-therapy. Suspending MSCs in hyaluronan commonly occurs in animals and humans, usually without supporting data.* Objective*. To elucidate the effects of different concentrations of hyaluronan on MSC growth kinetics.* Methods*. Using a range of hyaluronan concentrations, we measured MSC adherence and proliferation on culture plastic surfaces and a novel cartilage-adhesion assay. We employed time-course and dispersion imaging to assess MSC binding to cartilage. Cytokine profiling was also conducted on the MSC-secretome.* Results*. Hyaluronan had dose-dependent effects on growth kinetics of MSCs at concentrations of entanglement point (1 mg/mL). At higher concentrations, viscosity effects outweighed benefits of additional hyaluronan. The cartilage-adhesion assay highlighted for the first time that hyaluronan-primed MSCs increased cell attachment to cartilage whilst the presence of hyaluronan did not. Our time-course suggested patients undergoing MSC-therapy for OA could benefit from joint-immobilisation for up to 8 hours. Hyaluronan also greatly affected dispersion of MSCs on cartilage.* Conclusion*. Our results should be considered in future trials with MSC-therapy using hyaluronan as a vehicle, for the treatment of OA.

## 1. Introduction

Osteoarthritis is a degenerative disease characterised by degradation of cartilage and inflammation of the synovium [1]. Arthritic-degeneration and associated pain lead to reduced mobility, decreased economic contribution, and a significant healthcare burden of developed nations [2]. Conventional treatments of OA for middle-age sufferers are targeted at relieving pain using analgesics and nonsteroidal anti-inflammatories [3]. Others include intra-articular injection of exogenous preparations of hyaluronan (HA) as a viscosupplementation therapy [4]. End stage treatment of OA involves surgical realignment or total knee replacement (TKR) with artificial prostheses. Increasing lifespan has exacerbated the problem and widened the treatment gap for middle aged sufferers who are not suitable candidates for TKR [5].

Recently, adipose-derived mesenchymal stem cells (MSCs) have shown promise as an OA therapeutic. Numerous* in vitro* studies have demonstrated that MSCs can differentiate into mesodermal cell types that form cartilage, bone, and fat. However, differentiation of implanted cells* in vivo* is a rarely documented mode of action. Perhaps differentiated allogeneic cells are not immune privileged and would be removed by the host immune system. Paracrine effects, both anti-inflammatory and trophic, via the secretion of a complex mixture of cytokines are currently a more accepted and well-studied mode of action [6]. The trophic action of cytokines acts to stimulate and mobilise endogenous MSCs to repair and regenerate tissue.

MSCs can improve function and pain in patients suffering from knee OA with minimal adverse events. Patients show a consistent reduction in the size of cartilage defects by the regeneration and neoformation of hyaline-like cartilage [7]. Manufacture of cell therapeutics for OA may involve the use of HA, particularly as a vehicle. The rationale for this is that HA is a core component of the extracellular matrix, endogenously abundant in the knee joint [8, 9] and approved for the treatment of knee OA.

Suspending MSCs in HA is common practice in rodents [10] and larger animals [11, 12] and for the treatment of OA in humans [13, 14]. MSCs and HA are safe and are used independently in humans; however there are few studies which explore the effect of HA on MSC growth kinetics, function, and tissue binding, especially with respect to cartilage.

Therefore our study sought to address alterations of MSC-HA growth kinetics and whether changes would persist in HA primed cells both on plastic and on articular cartilage. We employed a novel cartilage disc assay in a time series to determine adherence time and assessed the physical dispersion of MSCs over cartilage shedding light on the biological relevance of the physiochemical phenomena of HA entanglement point. As the mode of action of MSCs is secretion-driven, the cytokine profile was measured to assess clinical feasibility of the MSC-HA combination.

## 2. Materials and Methods

### 2.1. MSC Growth Kinetics

*Adherence*. This experiment was undertaken on* standard* 96-well plates and CellBind 96-well plates (*high*-adherence; Corning, Australia) to test the effect of HA on different binding chemistries. Cells were seeded into 96-well plates at 5 × 10^3^ cells/well using a combination of media preparations (see Supplementary Figure  1 in Supplementary Material available online at http://dx.doi.org/10.1155/2016/9364213). A standard curve was seeded ranging from 1 × 10^3^ to 16 × 10^3^ cells/well. After 24 hours all wells were washed in PBS and 100 *μ*L of control media was added. A further 10 *μ*L of Cell Counting Kit-8 (water soluble tetrazolium salt, WST-8; CCK-8) reagent was added and following a four-hour incubation at 37°C, the absorbance measured at 450 nm (5 technical replicates; *n* = 3). Cell number was extrapolated from the standard curve using a trend line with a polynomial of two. Combinations of the media preparations (− +), (+ −), and (+ +) were compared back to cells grown in control media and seeded in control media (−  −) using a Mann-Whitney test (nonparametric test). 

*Proliferation.* This experiment was similar to the* adherence* protocol with minor differences. Cells were seeded into 96-well plates at 2 × 10^3^ cells/well (5 technical replicates; *n* = 3). The standard curve was seeded on the second day, 24 hours before the 3-day endpoint.

### 2.2. MSCs Kinetics on Equine Articular Cartilage Explants:* Ex Vivo *Cartilage Assay

All horses used in this study were due to be sacrificed at a commercial abattoir to be processed and sold as dog food (Kankool Pet Food, Australia). The fetlock joint from mature horses was cut out by sawing 15 cm below and above the joint. The fetlock was then shaved, scrubbed, and soaked in iodine bath for a minimum contact time of 5 minutes and then frozen for later use.

### 2.3. Cartilage Sectioning

The frozen fetlock joint was thawed at 37 degrees for 1-2 hours until the fetlock joint became flexible. Once thawed, the joint was disarticulated in a sterile hood (see Supplementary Figure 2). The third metacarpal was obtained from the fetlock joint and the distal end was soaked in PBS for 5 minutes. The articular cartilage was then perforated using a custom designed hollow cylindrical instrument measuring 6.45 mm in diameter equal to a* standard* 96-well plate. The perforated discs were then sliced off with a scalpel and stored in the correct orientation (joint side facing up) in a 96-well plate with 200 *μ*L of DMEM and frozen for later use.

### 2.4. Cartilage Adherence Time Course of MSCs

Cartilage discs were used to plug* ultra-low *adherence 96-well plates. Passage 2 cells were seeded onto the cartilage discs at a density of 5 × 10^3^ cells/disc and incubated at 37°C/5% CO_2_. At each time point (1, 2, 3, 4, 8, and 24 hours), cartilage discs were washed twice in PBS, fixed, and washed again. Cartilage discs were stained (see Supplementary Methods) and imaged using confocal microscopy.

### 2.5. HA Media Viscosity Assessment

Falling-ball viscometry was used to determine the viscosity (adapted from Eguchi and Karino, 2008 [15]) of HA media relative to control. Briefly, a 5 mL serological pipette was filled with prewarmed media. A biosilicate sphere was dropped and the time measured between two defined points. The data was expressed as a mean (5 technical replicates) flow-rate (millimetres/second) ± standard deviation.

### 2.6. MSC Dispersion on Cartilage with Increasing Concentration of HA

The media formulations used were control media and 0.5 mg/mL, 1 mg/mL, 2 mg/mL, 3 mg/mL, 4 mg/mL, and 5 mg/mL HA media. MSCs were seeded onto the cartilage discs at a density of 5 × 10^3^ cells/disc. Following a 24-hour incubation, cartilage discs were washed twice in PBS, fixed, and washed again. Cartilage discs were stained (see Supplementary Methods) and imaged using confocal microscopy.

### 2.7. MSC Adherence and Proliferation on Cartilage

MSCs were treated with either control or 1 mg/mL HA media for three days.* Ultra-low* adherence 96-well plates were plugged with cartilage discs. The conditions tested included MSCs grown in control and seeded in control (−  −), grown in control and seeded in 1 mg/mL HA media (− +), grown in 1 mg/mL HA media and seeded in control (+ −) (primed), and grown in 1 mg/mL HA media and seeded in 1 mg/mL HA media (+ +). 

*Adherence*. MSCs were seeded onto cartilage discs at a density of 5 × 10^3^ cells/disc. After 24 hours cartilage discs were removed to a new 96-well plate and washed twice with PBS. Fresh culture media (200 *μ*L) were added to discs followed by 20 *μ*L of CCK-8 and incubated for four hours at 37°C. Colour-developed wells were read at an absorbance of 450 nm (5 technical replicates; *n* = 3). 

*Proliferation.* This experiment was undertaken in the same way as the* adherence* experiment; however the cells were cultured for 3 days and seeded at a density of 2 × 10^3^ cells/disc.

### 2.8. Secretome Analysis

Conditioned media were collected from every flask in this study (*n* = 3), centrifuged at 5000 ×g for 5 minutes, and stored at −80°C. Filtrates (50 *μ*L) were analysed using both the Bio-Plex Pro-Human Cytokine 27-Plex and Bio-Plex Pro-Human Cytokine 21-Plex assay (Bio-Rad, USA), according to the manufacturer's instructions (see Supplementary Methods).

## 3. Results

### 3.1. MSCs Grown in Control Media and Seeded in HA Media

**Figure 1:**
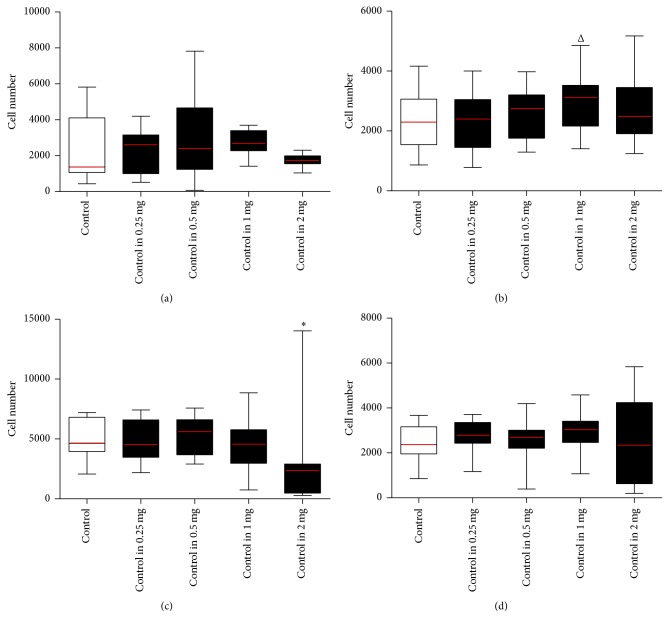
MSCs grown in control media and seeded in HA media. MSCs expanded until passage 2 in control media. Cells from flask (A) were then stripped and cell suspensions were counted using the standard enumeration technique. Cells were seeded into 96-well plates in either control media (control) (−  −) or HA media (− +) containing a series of concentrations ranging from 0.25 to 2 mg/mL of HA to make up the five conditions (*x*-axis). Conditions were tested using five technical replicates per 96-well plate and run in biological triplicate (*n* = 3). Wells were assayed at the endpoint using Cell Counting Kit-8 with standard curves run on every 96-well plate seeded 24 hours prior to endpoint and absorbance read at a wavelength of 450 nm (*y*-axis). All conditions were compared back to the control using a *t*-test (^Δ^ *p* value = 0.07, ^*∗*^ *p* value < 0.05). (a) MSCs seeded in* standard* 96-well plates for 24 hours (adherence), (b) MSCs seeded in* standard* 96-well plates for three days (proliferation), (c) MSCs seeded in* high*-adherence 96-well plates for 24 hours (adherence), and (d) MSCs seeded in* high*-adherence 96-well plates for three days (proliferation) (see Supplementary Table 1).

HA concentrations were compared to control, that is, (−  −) versus (− +). In* standard* 96-well plates, the highest cell proliferation was observed for 1 mg/mL HA (*p* < 0.07). In* high-*adherence 96-well plates the 2 mg/mL HA decreased adherence by 45% (*p* < 0.05) (Figure 1).

### 3.2. MSCs Grown in HA Media and Seeded in Control Media (Priming)

**Figure 2:**
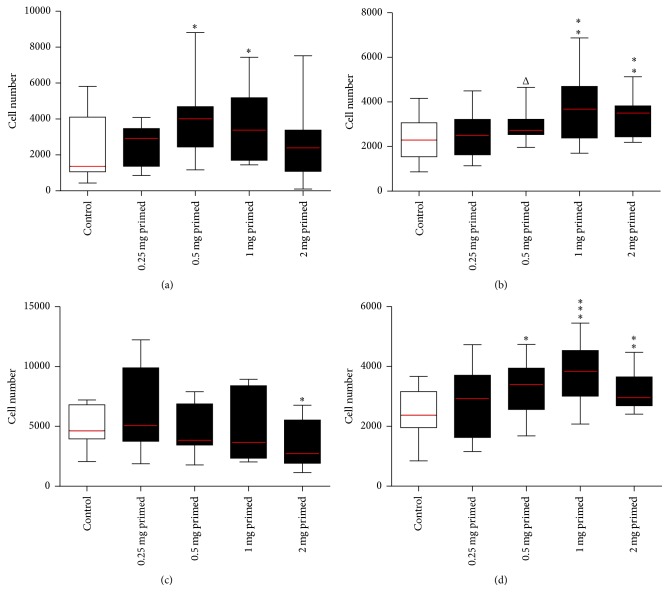
MSCs grown in HA media and seeded in control media (primed). MSCs expanded until passage 2 in control media. Cells were then stripped and cell suspensions were counted using the standard enumeration technique. Cells grown control media, flask (A), were seeded in control media (control) (−  −) and MSCs grown in flasks (B–E) HA media were seeded into 96-well plates in control media (HA primed) (+ −), to make up the five conditions (*x*-axis). Conditions were tested using five technical replicates per 96-well plate and run in biological triplicate (*n* = 3). Wells were assayed at the endpoint using Cell Counting Kit-8 with standard curves run on every 96-well plate seeded 24 hours prior to endpoint and absorbance read at a wavelength of 450 nm (*y*-axis). All conditions were compared back to the control using a *t*-test (^Δ^ *p* value = 0.1, ^*∗*^ *p* value < 0.05, ^*∗∗*^ *p* value < 0.01, and ^*∗∗∗*^ *p* value < 0.001). (a) MSCs seeded in* standard* 96-well plates for 24 hours (adherence), (b) MSCs seeded in* standard* 96-well plates for three days (proliferation), (c) MSCs seeded in* high*-adherence 96-well plates for 24 hours (adherence), and (d) MSCs seeded in* high*-adherence 96-well plates for three days (proliferation) (see Supplementary Table 1).

MSCs grown in HA media (B–E) were compared to control (−  −) versus (+ −). In* standard* 96-well plates, HA primed conditions showed peaked adherence for 0.5 mg/mL and 1 mg/mL HA (*p* < 0.05). Inversely, in* high-*adherence 96-well plates, adherence decreased with increasing concentration of HA and significantly for 2 mg/mL HA (*p* < 0.05). However in* high-*adherence 96-well plates, proliferative effects of HA priming recovered with 0.5, 1, and 2 mg/mL HA significantly (*p* < 0.05, <0.001, and <0.01, resp.) increased (Figure 2).

### 3.3. MSCs Grown in HA Media and Seeded in HA Media

**Figure 3:**
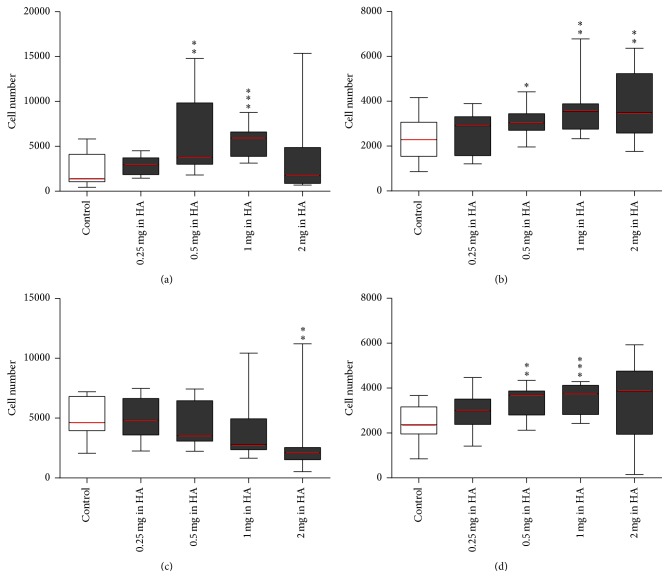
MSCs grown in HA media and seeded HA media. MSCs expanded until passage 2 in control media. Cells were then stripped and cell suspensions were counted using the standard enumeration technique. Cells grown control media, flask (A), were seeded in control media (control) (−  −) and MSCs grown in flasks (B–E) HA media were seeded into 96-well plates in HA media with the same concentration of HA (+ +) ranging from 0.25 to 2 mg/mL of HA to make up the five conditions (*x*-axis). Conditions were tested using five technical replicates per 96-well plate and run in biological triplicate (*n* = 3). Wells were assayed at the endpoint using Cell Counting Kit-8 with standard curves run on every 96-well plate seeded 24 hours prior to endpoint and absorbance read at a wavelength of 450 nm (*y*-axis). All conditions were compared back to the control using a *t*-test (^*∗*^ *p* value < 0.05, ^*∗∗*^ *p* value < 0.01, and ^*∗∗∗*^ *p* value < 0.001). (a) MSCs seeded in* standard* 96-well plates for 24 hours (adherence), (b) MSCs seeded in* standard* 96-well plates for three days (proliferation), (c) MSCs seeded in* high*-adherence 96-well plates for 24 hours (adherence), and (d) MSCs seeded in* high*-adherence 96-well plates for three days (proliferation) (see Supplementary Table 1).

MSCs grown in HA media (B–E) were compared to control (−  −) versus (+ +). In* standard* 96-well plates, adherence showed a dose-dependent trend with a peak at 1 mg/mL HA (*p* < 0.001) and decreased in 2 mg/mL HA. Similarly proliferation with 0.5, 1, and 2 mg/mL HA increased but peaked at 1 mg/mL HA (*p* < 0.05, <0.01, and <0.01, resp.). Inversely in* high-*adherence 96-well plates, adherence decreased with increasing concentration of HA and significantly for 2 mg/mL HA (*p* < 0.01). However proliferation again increased with 0.5 and 1 mg/mL HA (*p* < 0.01, <0.001, resp.) (Figure 3).

### 3.4. Time Course of MSC Grown on Cartilage

**Figure 4:**
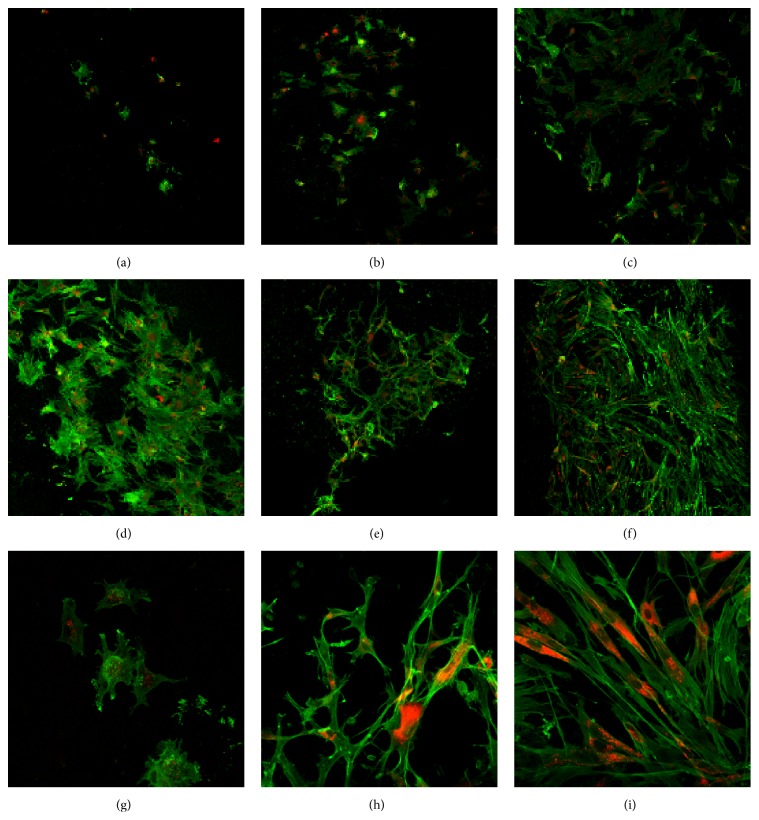
Cartilage adherence time course of MSCs. MSCs were cultured until the second passage before use in this experiment. Prior to seeding cells for the time course, the monolayer was washed in PBS and then stained using CM-DiI (orange colour) membrane dye. Cartilage discs were used to plug the bottom of a 96-well plate in the correct orientation. Cells were then stripped using TrypLE, counted using the standard enumeration technique, and then seeded onto the cartilage discs at a density of 5 × 10^3^ cells/disc and the 96-well plate was then incubated at 37°C and 5% CO_2_. At each of the time points (1, 2, 3, 4, 8, and 24 hours), the cartilage discs were washed in PBS and then fixed in 4% paraformaldehyde and then washed in PBS. Cartilage discs with attached cells were then permeabilised in 0.1% Triton X-100, washed in PBS, and blocked in 1% Bovine serum albumin in PBS before being stained with F-actin-specific Alexa Fluor 488-phalloidin (green). All images were taken using the OLYMPUS FLUOVIEW FV1000 IX81 inverted confocal microscope. (a) One-hour time point at 10x magnification, (b) two-hour time point at 10x magnification, (c) three-hour time point at 10x magnification, (d) four-hour time point at 10x magnification, (e) eight-hour time point at 10x magnification, (f) 24-hour time point at 10x magnification, (g) one-hour time point at 40x magnification, (h) eight-hour time point at 40x magnification, and (i) 24-hour time point at 40x magnification.

*Ultra-low* adherence 96-well plates were plugged with cartilage discs and MSCs seeded in control media to assess adherence times from one to 24 hours (Figure 4). At the one-hour time point (a), only a handful of cells adhered to cartilage. At higher magnification (g) MSCs appeared to be spherical with extensions clearly visible. From three to four hours (c-d), MSCs covered the surface with overlapping flat cell bodies. Overlapping between cells (e) was greatly reduced by eight hours and cell began to display (h) spindle morphology. At 24 hours (f) MSCs adhered to cartilage and dissipating membrane stain indicated possible doubling; (i) typical fibroblastic-like MSC morphology and organisation can be observed.

### 3.5. MSC Dispersion on Cartilage with Increasing Concentration of HA and Viscosity of Media Formulations

**Figure 5:**
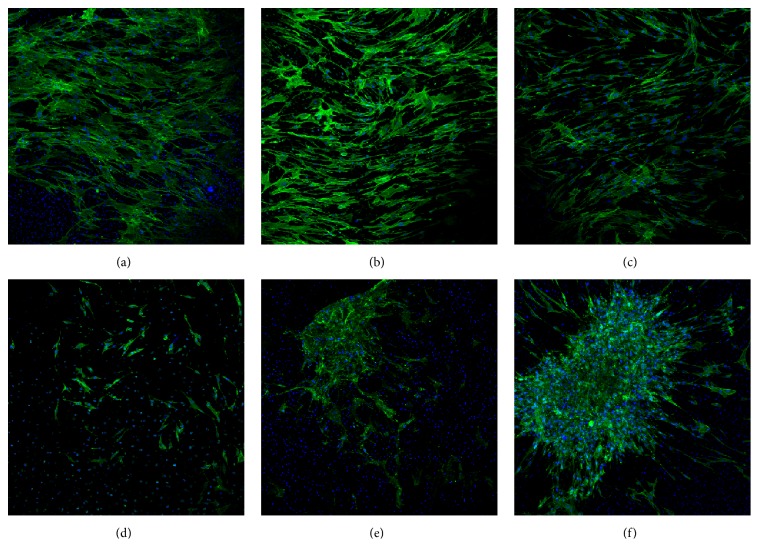
MSC dispersion on cartilage with increasing concentration of HA. MSCs were cultured until the second passage before use in this experiment. Cartilage discs were used to plug the bottom of a 96-well place in the correct orientation. Media formulations were then added to each well. The media formulations used were (a) 0.5 mg/mL HA media, (b) 1 mg/mL HA media, (c) 2 mg/mL HA media, (d) 3 mg/mL HA media, (e) 4 mg/mL HA media, and (f) 5 mg/mL HA media. MSCs were then stripped using TrypLE, counted using standard enumeration technique, and then seeded onto the cartilage discs at a density of 5 × 10^3^ cells/disc. The 96-well plate was then incubated for 24 hours at 37°C and 5% CO_2_. After 24 hours the cartilage discs were washed in PBS and then fixed in 4% paraformaldehyde. Cartilage discs were washed in PBS and then stained in using Hoechst 33342 (blue) and again washed five times in PBS. Cartilage discs were then permeabilised in 0.1% Triton X-100, washed in PBS, and blocked in 1% Bovine serum albumin in PBS before being stained with F-actin-specific Alexa Fluor 488-phalloidin (green). All images were taken using the OLYMPUS FLUOVIEW FV1000 IX81 inverted confocal microscope at 10x magnification.

*Ultra-low* adherence 96-well plates were plugged with cartilage discs and MSCs were seeded, suspended in either control or HA media (0.5–5 mg/mL HA). At lower concentrations of HA, MSCs uniformly dispersed across the cartilage surface. As concentration increased to 3 mg/mL dispersion was maintained but with reduction in adhered cells. Singular colonies were observed at highest concentrations 4 mg/mL and 5 mg/mL HA (Figure 5).

The flow-rate of the sphere in the falling-ball test was measured to show viscosity of HA media formulations relative to control (see Supplementary Figure 3). The control displayed the highest flow-rate, which decreased with increasing concentrations of HA. The flow-rate for 0.5 mg/mL and 1 mg/mL HA was 57% and 51% of the control, respectively. Flow-rate of 2 mg/mL HA dropped to 20% of control and then a plateau was observed for higher concentrations.

### 3.6. MSC Adherence and Proliferation on Cartilage

**Figure 6:**
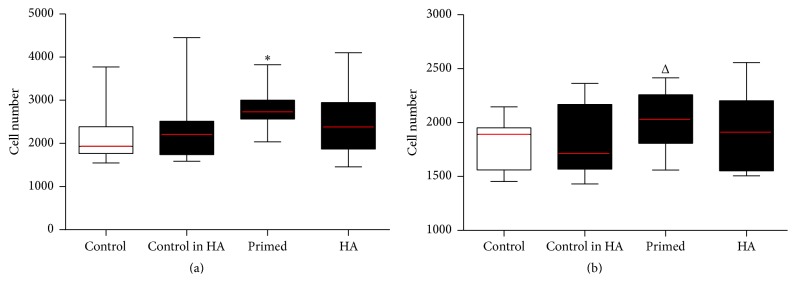
Treated MSCs seeded onto articular cartilage explants for 24 hours and three days. MSCs expanded until passage 2 in control media. On the third day flasks were treated with either control media or 1 mg/mL HA media for another three days. Cells were then stripped and cell suspensions were counted using the standard enumeration technique. Cells were seeded into* ultra-low* adherence 96-well plates plugged with* articular cartilage explants* in the correct orientation. Conditions (*x*-axis) tested were cells grown in the control flask and seeded in control media (control) (−  −), grown in control media and seeded in 1 mg/mL HA media (control in HA) (− +), grown in 1 mg/mL HA media and seeded in control media (primed) (+ −), and grown in 1 mg/mL HA media and seeded in 1 mg/mL HA media (HA) (+ +) and articular cartilage explants alone (no cells seeded to serve as a blank absorbance). Conditions were tested using five technical replicates per 96-well plate and run in biological triplicate (*n* = 3). Wells were assayed at the endpoint using Cell Counting Kit-8 and absorbance of the resulting media alone read at a wavelength of 450 nm (*y*-axis). All conditions were compared back to the control using a *t*-test (Δ = 0.19, ^*∗*^ *p* value < 0.05). (a) MSCs seeded for 24 hours (adherence) into* ultra-low* adherence 96-well plates plugged with articular cartilage explants and (b) MSCs seeded for three days (proliferation) into* ultra-low* adherence 96-well plates plugged with articular cartilage explants.

The 1 mg/mL HA formulation was tested for all conditions (Figure 6).* Ultra-low* adherence 96-well plates were plugged with cartilage discs and cells seeded at either 5 × 10^3^ cells (*adherence*) or 2 × 10^3^ cells (*proliferation*) per well. In the adherence experiment, a peak was observed in the primed (+ −) condition (*p* < 0.05). A similar peak was observed for the primed condition in the proliferation experiment (*p* = 0.1).

### 3.7. HA Media Alter the Cytokine Secretion Profile of MSCs

**Figure 7:**
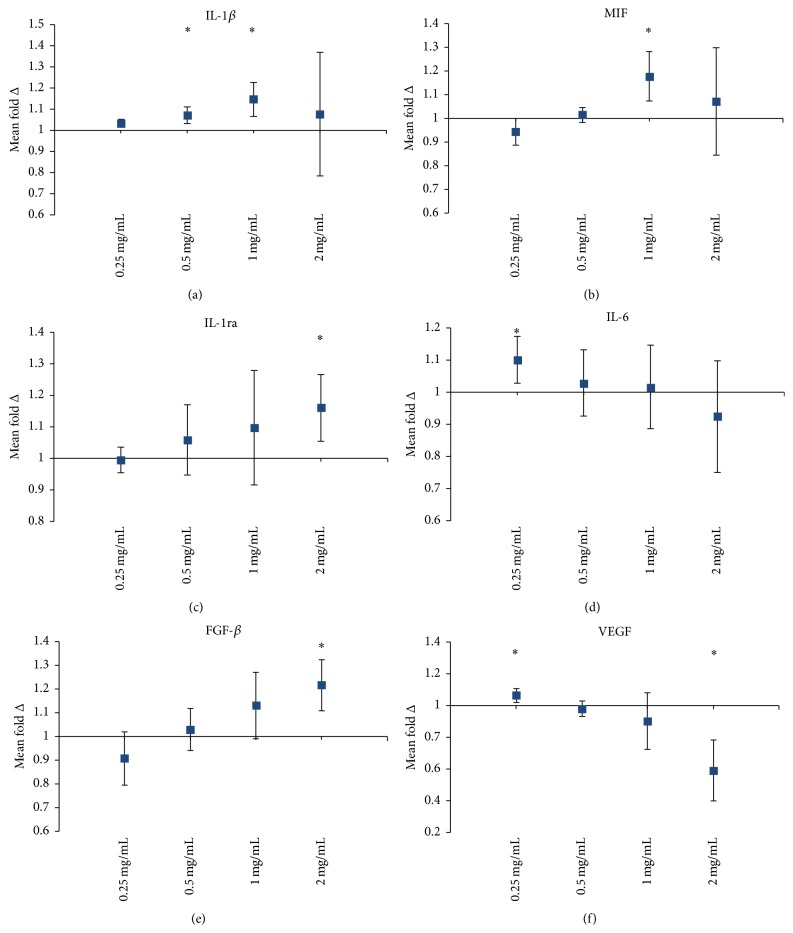
Fold change of cytokine secretion by MSCs with HA treatment. Conditions were all cultured in either control media or HA media for three days after the media change. Conditions (*x*-axis) were grown in flasks (B–E) HA media, ranging from 0.25 to 2 mg/mL of HA. These were all compared back to the control condition (cells grown control media, flask (A)). Average fold change in fluorescence (*n* = 3) ± upper and lower confidence intervals at 95% (*y*-axis). Cytokines with a fold change less than one indicate a decrease with HA media treatment; a fold change greater than one indicates an increase in the cytokine with HA treatment (^*∗*^cytokines with a fold change clear of the axis at 1 reported as significant). Interleukin-1*β* (IL-1*β*), macrophage migration inhibitory factor (MIF), Interleukin-1 receptor antagonist (IL-1ra), Interleukin-6 (IL-6), fibroblast growth factor-basic (FGF-*β*), and vascular endothelial growth factor (VEGF). See Supplemental Data for complete list of significantly changed cytokines and numerically tabulated summary.

MSCs were treated for three days in either control or HA media formulations. Changes in concentration were observed in 28 of the 48 measured cytokines (see SF  4–7 and Supplementary Table 2). MSCs treated with 1 mg/mL HA increased secretion for Interleukin-1*β* (IL-1*β*) and macrophage migration inhibitory factor (MIF) (Figure 7). Anti-inflammatory cytokine Interleukin-1 receptor antagonist (IL-1ra) secretion increased dose-dependently and was significant at 2 mg/mL HA. Interleukin-6 (IL-6), a dual role cytokine, increased in the 0.25 mg/mL HA concentration but then decreased with increasing concentration of HA. Fibroblast growth factor-basic (FGF-*β*) increased dose-dependently and was significant at 2 mg/mL HA. Vascular endothelial growth factor (VEGF) decreased with increasing concentration of HA and was significant at 2 mg/mL HA.

## 4. Discussion

Biological therapeutics such as MSC therapy are gaining acceptance for the treatment of inflammatory conditions, including musculoskeletal ailments such as knee osteoarthritis (OA). This is most likely due to the well documented treatment gap for middle-age sufferers of OA who are not candidates for total knee arthroplasty [5]. The safety of allogeneic MSC therapy will continue to drive the universal donor model because an “off-the-shelf” therapeutic is desirable for scalability and cost. The manufacture of cell therapeutics for the treatment of OA will likely involve the use of agents that can enhance MSC function. The use of HA is attractive as an adjunct, as it is a core component of the extracellular matrix, endogenously abundant in the knee joint [8, 9] and approved for the treatment of OA. It is important to understand growth kinetics of MSCs with respect to HA. Already there have been case reports [16] and randomised, double-blind, placebo controlled studies [14] where MSCs were suspended in HA either as a vehicle or as a combination for the treatment of OA. These studies did not document any exploration of positive or negative interactions between MSCs and HA [17]. Therefore we tested different concentrations of HA in culture media to assess adherence and early proliferation kinetics of MSCs on both plastic culture surfaces and equine articular cartilage explants in an* ex vivo* model, as a way to simulate early cell behaviour following an intra-articular injection of this combination. Also, we hypothesised that the high viscosity of HA may interfere with cell binding. To avoid this issue, we explored whether MSCs could be primed with HA to improve growth kinetics or adherence. We employed a novel cartilage disc assay in a time series to determine MSC adherence to cartilage. In the same cartilage assay the physical dispersion of MSCs was assessed using a concentration series of HA media over the cartilage shedding light on the biological relevance of the entanglement point of HA chains.

An allogeneic preparation of MSCs and HA may be developed in a number of ways, with HA only present in the final cellular product, used as a priming agent during culture but not in the final formulation or both. Here we tested three representative conditions and found that cells grown in standard culture and seeded in HA (− +) show some increases in adherence and proliferation. However, greater differences were observed in the (+ −) and (+ +) conditions, where HA as a culture supplement exhibited dose-dependent increases in cell number. It is well known that HA molecules behave in solution as highly hydrated randomly kinked coils, which start to entangle at concentrations of less than 1 mg/mL (entanglement point) [18]. The peak seen in the HA dose required for optimal cell attachment is centred on this entanglement point of the HA chains. In this study, for the first time we have attributed biological significance to the physiochemical phenomena of the entanglement point seen in HA chains. At this concentration of HA, all HA molecules are connected via a meshwork and behave like a weak and elastic gel which mimics properties of soft tissue [19].

In contrast to the standard plates, the* high-*adherence plates decreased the number of adherent cells in the presence of HA and for the HA primed cells. According to the manufacturer, the surface is treated (nonbiologically) to improve cell attachment by incorporating more oxygen into the cell culture surface by increasing surface area and rendering it more hydrophilic. Increasing concentrations of HA decreased the number of attached cells within the first 24 hours significantly. HA is a regulator of water homeostasis in the body [8]; therefore it is reasonable to suggest HA interfered with the treated surface, an interference which persisted in the primed conditions.

The constraint of viscosity made it unclear whether increasing HA concentration further would amplify the effects seen in early time points. Viscosity-induced heterogeneity can be seen in the variance of the ranges observed for the 2 mg/mL HA concentration across all the growth kinetics experiments when cells were seeded in HA. Previous investigations of porcine bone marrow-derived MSCs showed using 4 mg/mL HA (*M*_*W*_ 800 KDa) was optimal at 7 days [20] but no changes in two days. It is counterintuitive to expect optimal growth kinetics at 7 days since HA half-life in the knee joint is reported to be less than 24 hours [21].

Our study was concerned with high molecular weight HA, as it is more applicable as an OA therapeutic [22]. The study of low molecular weight HA found no difference in cell adherence or proliferation between 0.5, 1, and 2 mg/mL concentrations in the early time points. Another adhesion study using the same high molecular weight HA also found a decrease in synovial MSC adhesion with increasing concentrations beyond the entanglement point [23]. We employed a modified falling-ball test to show relative changes in viscosity from control media. The biggest change in viscosity was seen at concentrations above the entanglement point, the flow-rate of which decreased by 31% between 1 and 2 mg/mL concentrations and then a plateau. This further explains decreases seen in cell attachment for concentrations higher than 1 mg/mL as a viscosity constraint in the early phase.

Increased attachment and proliferation on culture surfaces enabled the initial experiments to be performed under controlled conditions but may not reflect cartilage binding. We therefore employed a novel equine-derived cartilage explant culture model* ex vivo* in an attempt to better mimic true architecture of the target tissue in OA, cartilage. Shimaya et al. had shown previously that synovial MSCs will increase adherence to an osteochondral defect with 10 mM magnesium* ex vivo* [24]. However, inconsistencies can be observed in defects created and further limitations of subjectivity can arise when using an image analysis technique to quantify cell adherence. Baboolal et al. utilised a similar technique with osteochondral plugs embedded in agarose to test MSC adherence to cartilage. However, MSCs were labelled with iron-oxide microparticles and semiquantitative image analysis techniques were used to report cell adhesion [23]. Herein we have described a reproducible, consistent, and quantitative method for cell attachment to biologically inactive cartilage in isolation. This enabled the testing of various cell and HA formulations without any modulatory signalling from the cartilage because it was not viable. Some considerations for our technique revolved around the plug integrity in the well; that is, if the cartilage disc curled and the well no longer sealed, the replicate was discarded. For this reason, all conditions were run using five technical replicates. The use of* ultra-low* adherence plates and repeated washing limited nonspecific binding of cells to plastic and therefore gave a true indication of cell adherence to the cartilage.

Growth kinetics on the cartilage surface employed the optimal concentration of 1 mg/mL HA for all the conditions on* ultra-low* adherence plates plugged with the cartilage discs as the new surface. Interestingly, if the cells were seeded in the presence of HA (− + and + +), cell adherence did not improve which suggests the HA may interfere with MSCs binding to cartilage. Only the primed MSCs adhered (*p* < 0.05) and proliferated (*p* = 0.1) more than the control. Therefore it is reasonable to expect that the use of HA as a vehicle for MSCs during intra-articular injection for knee OA will interfere with cell attachment to cartilage. Indeed this was consistent with previous investigations, where high molecular weight HA in OA-derived synovial fluid interfered with MSCs binding to cartilage, an effect which could be overcome by hyaluronidase (enzyme used to break down HA) treatment [23]. Furthermore it was shown in a caprine model using a collagen II-induced arthritis that the half-life of HA was an average of 11.5 hours and 20.8 hours in the nonchallenged knees [25].

We showed MSCs substantially adhere after three hours but do not regain fibroblastic morphology until eight hours after seeding. For surgeons injecting allogeneic MSCs into humans, this may indicate the need for an extended unweighted rest period of 8–24 hours after injection to allow MSCs to thoroughly adhere to the cartilage in the joint. Although our model does not take into account the internal pressures of the knee joint, endogenous HA concentration or the catabolic milieu of an OA affected knee. Previous investigations of cell attachment to human arthritic cartilage showed synovial MSCs can adhere within 10 minutes [24]; however the cell dose used was more than 200 times greater per square millimetre of cartilage and lacked washing steps to account for loosely bound cells. In light of this, the model cannot take into account internal shearing forces of the joint which may contribute to cell detachment. Also no effort was made to show morphology of cells was fibroblastic and therefore they may have been in the flat bodied and in the initial attachment phase observed in this study. Furthermore, OA-derived synovial fluid was shown to inhibit MSC binding to cartilage [23]; thus an extended rest period beyond 24 hours may be required for thorough adhesion.

Concentration of HA as a vehicle for intra-articular injection of MSCs is important for dispersion and internal cartilage coverage. The dispersion assay with increasing concentration of HA indicated the higher (4 and 5 mg/mL HA) concentrations would form single aggregate colonies. A randomised, double-blind, controlled study which used intra-articular injection of allogeneic bone marrow-derived MSCs with HA as a vehicle at a concentration of 10 mg/mL showed a reduction in pain in patients with OA changes using a visual analogue scale [14]. However, only some patients had increased meniscal volume, which may be* de novo* tissue regeneration. According to our dispersion data, it is likely that the injected cell suspension did not disperse throughout the joint. Furthermore, the endogenous concentration of HA found in postmortem synovial fluid was reported at 1–4 mg/mL of HA [26] and this can decrease with joint pathologies as reported by Dahl et al. to 0.17–1.32 mg/mL [27]. It is unclear if the lack of dispersion and therefore close proximity of MSCs would have an effect on tissue regeneration or migration of MSCs* in vivo*. However, MSCs grown in hanging drop culture as spheroids express higher levels of anti-inflammatory TNF alpha stimulated gene 6 (TSG-6) and stanniocalcin-1 (STC-1) compared to monolayer culture [28] which suggests altered immunomodulatory capacity of MSCs.

Culturing MSCs with HA can alter the cytokine secretion profile. Investigations of the secretome of MSCs when combined with HA have been shown to increase the secretion of macrophage migration inhibitory factor (MIF) [29]. MIF secretion by MSCs in this study peaked significantly only for the 1 mg/mL treated cells. MIF is considered to be a proinflammatory mediator especially in OA and acts in local tissue to increase neutrophil and macrophage migration to regions of inflammation. Our previous investigations also showed increased secretion of MIF by MSCs cultured with HA [29]. Vascular endothelial growth factor (VEGF) decreased with increasing concentration of HA and significantly for the 2 mg/mL concentration. This was consistent with our previous investigations of HA and MSC coculture [29]. VEGF may play an active role in the pathogenesis of OA. Ludin et al. found that if exogenous VEGF was injected into the knees of mice, the joint presented with synovial hyperplasia and cartilage degradation as typically seen in OA disease [30]. Thus a consistent decrease in the secretion of VEGF by MSCs in the presence of HA may suggest therapeutic synergy between the two.

Basic fibroblast growth factor (FGF-*β*) increased with increasing concentrations of HA. FGF-*β* is a potent mitogen on MSCs and the increased secretion may help to explain the increase in proliferative capacity of the cells [31, 32]. HA may therefore have an agonistic role in potentiating the secretion of FGF-*β* by MSCs but this has not been shown. Additionally, an increase in secretion of FGF-*β* may contribute to increase therapeutic efficacy of MSCs for the treatment of OA. A cartilage defect model in rabbit showed exogenous human FGF-*β* alone could potentiate articular cartilage resurfacing within 6 weeks of the injury [33].

## 5. Conclusion

Allogeneic preparations of MSCs will become an increasingly attractive therapeutic in the treatment of knee OA. These preparations are increasingly seen to be tested with HA as a vehicle without consideration for the likely interactions. Here we have shown HA can have profound dose-dependent effects on early growth kinetics of MSC and these effects were founded on the physiochemical phenomena of the HA entanglement point. The use of our* ex vivo* cartilage model further emphasises the limitation of* in vitro* experimentation but also highlighted for the first time that HA primed MSCs can increase cell attachment to cartilage and that the presence of HA did not. Our time course study also suggests patients undergoing MSC therapy for knee OA could benefit from being immobilised and unweighted for 8 hours after injection to allow MSCs to thoroughly adhere in the joint. Additionally, concentration of HA was also shown to greatly affect the dispersion of MSCs. These factors should be considered in future trials with respect to the culture media for MSCs and where HA is being considered as a component of vehicle for the treatment of OA.

## Supplementary Material

The supplementary material contains extended methods, additional figures and numerical tabulations of the graphs used in the study. These are provided as a reference for the reader for ease of interpretation of the study.
