# Supplementary material for: Priming Adipose-Derived Mesenchymal Stem Cells with Hyaluronan Alters Growth Kinetics and Increases Attachment to Articular Cartilage
Source: Stem Cells Int. 2016 Feb 15;2016:9364213. doi: 10.1155/2016/9364213 (PMC4770159; doi:10.1155/2016/9364213)
Supplement: Supplementary file 1 — The supplementary material contains extended methods, additional figures and numerical tabulations of the graphs used in the study. These are provided as a reference for the reader for ease of interpretation of the study. [file 9364213.f1.docx]

Supplemental Material

# Methods

Isolation of human adipose-derived mesenchymal stem cell (MSC) populations

This research was approved by the Macquarie University human research ethics committee (Ref #: 5201100385). Human lipoaspirate was obtained from patients undergoing elective cosmetic liposuction surgery. The lipoaspirate was digested as previously described. Briefly, 200mL of fresh lipoaspirate was mixed and enzymatically digested in pre-warmed (37°C) saline containing 0.5 mg/mL collagenase (Lomb Scientific, USA). The lipoaspirate was then incubated in a 37°C water bath for 30 minutes and mixed periodically to circumvent layer separation. The digested lipoaspirate was then passed through an 800 μm mesh to exclude undigested tissue clumps. Finally, the suspension was centrifuged at 1500 x g for 5 minutes to obtain the pelleted stromal vascular fraction cells (SVF) and floating adipocytes.

Propagation of adherent adipose-derived mesenchymal stem cells

To obtain a population of adherent MSCs, the SVF pellet was transferred into three T175cm^2^ flasks containing Standard culture media (control media) which consisted of Dulbecco’s Modified Eagle Medium (DMEM; Invitrogen, USA) supplemented with 10% foetal bovine serum (FBS; Bovogen, Australia) and 1% Penicillin-Streptomycin solution (Invitrogen, USA). Flasks were incubated for 72 hours at 37°C with 5% carbon dioxide. To prevent iron toxicity to adherent MSCs; the flasks were washed using DMEM with no additives to wash away non-adherent cells and the media replaced with fresh standard culture media. Once the monolayer of MSCs reached 80% confluency, cells were passaged with enzymatically using TrypLE express (Invitrogen, USA) and then re-suspended in standard culture media. Cells from each flask were then transferred to three new T175cm^2^ flasks now termed passage 1. Media changes were performed every 72 hours. Once all the flasks were confluent, the cells were stripped, counted and frozen in 90% FBS and 10% DMSO firstly in isopropanol containing vessel for gradual cooling in a -80°C freezer and then transferred to liquid nitrogen for long term storage.

Standardised enumeration

Standardised enumeration of cells was achieved with Tru-Count tubes (Becton Dickinson, USA) containing sheath fluid (isoflow; Becman Coulter, USA) and nucleic acid dyes; propidium iodide (10 µg/mL; Sigma, USA), Syto11 (1 µM; Invitrogen, USA) and a defined bead number. The combination of a charged (propidium iodide; Pi) and a cell permeate (Syto11) dye allows for discriminant cell population gating, which are based on the principal of Pi membrane exclusion and thus viability can be calculated from defined bead populations run as standards. Samples were all run on FACSCalibur flow cytometer (Becton Dickinson, USA).

MSC growth kinetics

MSCs were all used from frozen preparations at passage one and were cultured until the second passage before use in this experiment. The flasks were left in control media for another three days after seeding them in the second passage. On the third day each of the flasks were treated with a range of media formulations supplemented with high molecular weight HA (*M_W_ 2600 KDa)* (Lifecore Biomedical, USA) for another three days (see supplemental figure 1). These were control media, control + 0.25 mg/mL HA (0.25 mg HA media), control + 0.5 mg/mL HA (0.5 mg HA media), control + 1 mg/mL HA (1 mg HA media) and control + 2 mg/mL HA (2mg HA media). Another three days after each of the flask had been treated, the conditioned media from each was collected, centrifuged at 5000 RCF for 5minutes and frozen for later analysis. The cells were then stripped using TrypLE, made up in a final volume of 5 mL of control media and then the cells were counted using the standard enumeration technique.

*Adherence.* This experiment was undertaken on *standard* 96-well plates and CellBind 96-well plates (*High-*adherence*;* Corning, Australia) to test the effect of HA on different binding chemistries. Once a cell number was achieved for each of the flasks, the cells would be seeded into a 96-well plate at 5x10^3^ cells/well using a combination of media preparations (see Supplemental figure 1). Alongside these test wells, a standard curve was seeded ranging from 1x10^3^ to 16x10^3^ cells/well. After 24 hours all the wells were washed twice with PBS and 100 µL of fresh control media added. A further 10 µL of CCK-8 reagent was added and following a four hour incubation at 37°C, the absorbance measured at 450 nm. An average of five technical replicates for each media preparation per biological replicate (*n=3*). Cell number was extrapolated from the standard curve. All combinations of the media preparations ***(- +)***, ***(+ -)*** and ***(+ +)*** were compared back to the cells grown in control media and seeded in control media ***(- -)*** using a t-test (two-sided). All calculations were done using Microsoft excel 2013.

*Proliferation.* This experiment was undertaken on *standard* 96-well plates and *high-*adherence 96-well plates. This arm of the experiment was undertaken in a similar way to the adherence experiment. Once the cell number was achieved for each of the flasks, the cells were seeded into a 96-well plate at 2x10^3^ cells/well using a combination of media preparations (see Supplemental figure 1). The standard curve would be seeded 24 hours (on the second day) before the endpoint At the 3 day time point, all the wells were washed twice in PBS, replaced with 100 µL of fresh control media followed by the addition of 10 µL of CCK-8 reagent and at the conclusion of a four hour incubation at 37°C, the absorbance measured at 450 nm. An average of five technical replicates for each media preparation per biological replicate (*n=3*). Cell number was extrapolated from the standard curve. All combinations of the media preparations ***(- +)***, ***(+ -)*** and ***(+ +)***, were compared back to the cells grown in control media and seeded in control media ***(- -)*** using a t-test (two-sided). All calculations were done using Microsoft excel 2013.

MSCs kinetics on equine articular cartilage explants: *ex vivo* cartilage assay

All horses used in this study were due to be sacrificed at a commercial abattoir to be processed and sold as dog food (Kankool Pet Food, Australia). The fetlock joint from mature horses was cut out by sawing 15 cm below and above the joint. The fetlock was then shaved, scrubbed and soaked in iodine bath for a minimum contact time of 5 minutes and then frozen for later use.

Cartilage sectioning

The frozen fetlock joint was thawed at 37 degrees for 1-2 hours until the fetlock joint became flexible. Once thawed, the joint was dis-articulated in a sterile hood (see supplemental figure 2). The third metacarpal was obtained from the fetlock joint and the distal end was soaked in PBS for 5 minutes. The articular cartilage was then perforated using a custom designed hollow cylindrical instrument measuring 6.45 mm in diameter equal to a *standard* 96-well plate. The perforated discs were then sliced off with a scalpel and stored in the correct orientation (Joint side facing up) in a 96-well plate with 200 µL of DMEM and frozen for later use.

Cartilage adherence time course of MSCs

MSCs were used from frozen preparations at passage one. The cells were cultured until the second passage before use in this experiment. Prior to seeding cells for the time course, the monolayer was washed in PBS and then stained using CM-DiI (1 µM; life technologies, Australia) membrane dye made in PBS according to the manufacturer’s instructions. Cartilage discs were used to plug the bottom of an *ultra-low* adherence 96-well plate in the correct orientation. Cells were then stripped using TrypLE, counted using standard enumeration and then seeded onto the cartilage discs at a density of 5x10^3^ cells/disc and the plate then incubated at 37°C and 5% CO_2_. At each of the time points (1, 2, 3, 4, 8 & 24 hours), the cartilage disc was washed twice in PBS and then fixed in 4% paraformaldehyde for one hour and then washed in PBS five times. Cartilage discs with attached cells were then permeabilised in 0.1% Triton X-100 for five minutes, washed twice in PBS, blocked in 1% Bovine serum albumin in PBS for 20 minutes before being stained with F-actin specific Alexa Fluor 488-phalloidin (0.165 µM; life technologies, Australia) at room temperature, in the dark for 20 minutes and then washed thrice in PBS. Imaging of the cartilage discs was performed at the Macquarie University Microscopy Unit with the OLYMPUS FLUOVIEW FV1000 IX81 inverted confocal microscope (Olympus, Australia).

HA media viscosity assessment

Falling-ball viscometry was used to determine the viscosity (adapted from Eguchi and Karino, 2008 ^17^) of HA media relative to control media. Briefly, a 5 mL serological pipette with a diameter of 5.9 mm was filled with media pre-warmed to 37°C. A bio-silicate sphere, 3 mm in diameter was dropped and the time measured between two defined points with a known distance. A total of five replicates for each media were obtained. The data was expressed as a mean flow rate (millimetres per second) and the stand deviation as error bars.

MSC dispersion on cartilage with increasing concentration of HA

MSCs were used from frozen preparations at passage one. The cells were cultured until the second passage before use in this experiment. Cartilage discs were used to plug the bottom of an *ultra-low* adherence 96-well place in the correct orientation. Control media (200 µL) was then added to each well. The media formulations used were control media, 0.5 mg HA media, 1 mg HA media, 2 mg HA media, 3 mg HA media, 4 mg HA media or 5 mg HA media. MSCs were stripped using TrypLE, counted using standard enumeration and then seeded onto the cartilage discs at a density of 5x10^3^ cells/disc. The 96-well plate was then incubated for 24 hours at 37°C and 5% CO_2_. After 24 hours the cartilage discs were washed twice in PBS and then fixed in 4% paraformaldehyde for one hour. Cartilage discs were washed in PBS five times and then stained in 1 mL of Hoechst 33342 (2 µg/mL; life technologies, Australia) for five minutes and again washed five times in PBS. Cartilage discs with attached cells were then permeabilised in 0.1% Triton X-100 for five minutes, washed twice in PBS, blocked in 1% Bovine serum albumin in PBS for 20 minutes before being stained with F-actin specific Alexa Fluor 488-phalloidin (0.165 µM; life technologies, Australia) at room temperature, in the dark for 20 minutes and then washed thrice in PBS. Imaging of the cartilage discs was performed at the Macquarie University Microscopy Unit with the OLYMPUS FLUOVIEW FV1000 IX81 inverted confocal microscope (Olympus, Australia).

MSC adherence & proliferation on cartilage

MSCs were used from frozen preparations at passage one. The cells were cultured until the second passage before use in this experiment. The flasks were left in control media for another three days after the seed. On the third day the flasks were treated with either control or 1 mg/mL HA media for another three days. After the cells had been treated for 3 days, the plate containing cartilage discs was thawed. Enough discs were then transferred into *ultra-low* adherence plate in order to obtain 5 technical replicates per condition. The conditions tested in this assay followed the same matrix as shown in supplemental figure 1. These were MSCs grown in control media and seeded in control media ***(- -)***, grown in control media and seeded in 1 mg/mL HA culture media ***(- +)***, grown in 1 mg/mL HA media and seeded in control media ***(+ -)*** (primed) and grown in 1 mg/mL HA media and seeded in 1 mg/mL HA media ***(+ +)***.

*Adherence.* MSCs were seeded onto cartilage discs at a density of 5x10^3^ cells/disc. After 24 hours the cartilage discs were removed to a new 96-well plate and washed twice with PBS. Fresh culture media (200 µL) was added to the discs followed by 20 µL of CCK-8 reagent and then incubated for four hours. The cartilage discs were then removed from the colour developed well and the absorbance read at 450 nm. Data represents an average of 5 technical replicates and a minimum of 3 biological replicates. *Proliferation.* This experiment was undertaken in the same way as the adherence experiment, however the cells were cultured for 3 days and seeded at a density of 2x10^3^ cells/disc.

Secretome analysis

The conditioned media was collected from every flask in this study, centrifuged at 5000 x g for 5 minutes and stored at -80°C. Upon thawing, the samples were filtered through 0.2 mm Nanosep MF Centrifugal Devices with Bio-InertH Membrane (Pall Scientific, USA). Filtrates (50 μL) were analysed using both the Bio-Plex Pro Human Cytokine 27-plex and the Bio-Plex Pro Human Cytokine 21-plex assay (Bio-Rad, USA), according to the manufacturer’s instructions. The washing steps were performed using the Bio-Plex Pro II magnetic wash station and the data was acquired using the Bio-Plex 200 system with version 5.0 software (Bio-Rad, USA). The average fluorescence of each cytokine in the conditioned medium samples was calculated from four technical replicates in cultured MSC experiments. To account for biological variation in the secreted cytokines, the data was normalised to fold change in fluorescence of HA treated over control (*n=3*). The fold change was then averaged and graphed with the upper and lower confidence intervals set at 95% as error bars. Cytokines with a fold change less than one indicate a decrease in concentration with HA media treatment, a fold change greater than one indicates an increase in the cytokine with HA treatment. Any cytokines with a fold change clear of the axis at 1 were reported as significant (determined numerically).

# Figures and Tables

| *MSCs frozen at passage 1 were thawed*  *Cells are put into culture until confluent monolayer is achieved* | 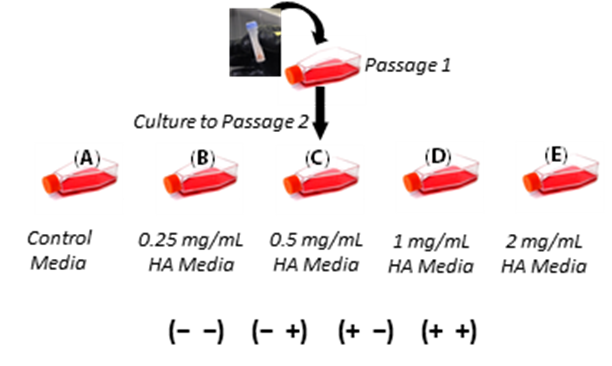 |
| --- | --- |
| *Cells passaged at a ratio of 1:3*  *After reaching passage 2, flasks are kept in control media for three days* |  |
| *Media is changed into either control media or media containing Hyaluronan for another three days* |  |
| *Cells are seeded into 96-well plates in a range of media formulations* |  |
| ***Legend:*** | *Cells grown in control media and seeded into test wells with control media*  **(− −)** |
|  | *Cells grown in control media and seeded into test wells with HA media*  **(− +)** |
|  | *Cells grown in HA media and seeded into test wells with control media (primed)*  **(+ −)** |
|  | *Cells grown in HA media and seeded into test wells with HA media*  **(+ +)** |
| **Supplemental figure 1 (SF 1) Experimental schematic and seeding conditions**  MSCs were cultured until passage 2. After passage all flask were kept in control media for three days. The media was then changed into Control media (flask A), 0.25 mg/mL HA media (flask B), 0.5 mg/mL HA media (flask C), 1 mg/mL HA media (flask D) and 2 mg/ml HA media (flask E). Following HA treatment, cells were then stripped, counted using the standard enumeration technique and then seeded into test wells in a range of media formulations to make up the different conditions. Conditions were always compared back to the control (flask A) which consisted of cells grown in control media and seeded into test wells in control media ***(- -)***. Cells grown in flask A were seeded into test wells using HA media ***(- +)***. Cells grown in flasks B-E were seeded into test wells using control media ***(+ -)*** (primed). Cells grown in flasks B-E were seeded into test wells using HA media at the same concentration of HA used to treat ***(+ +)***. | |

| 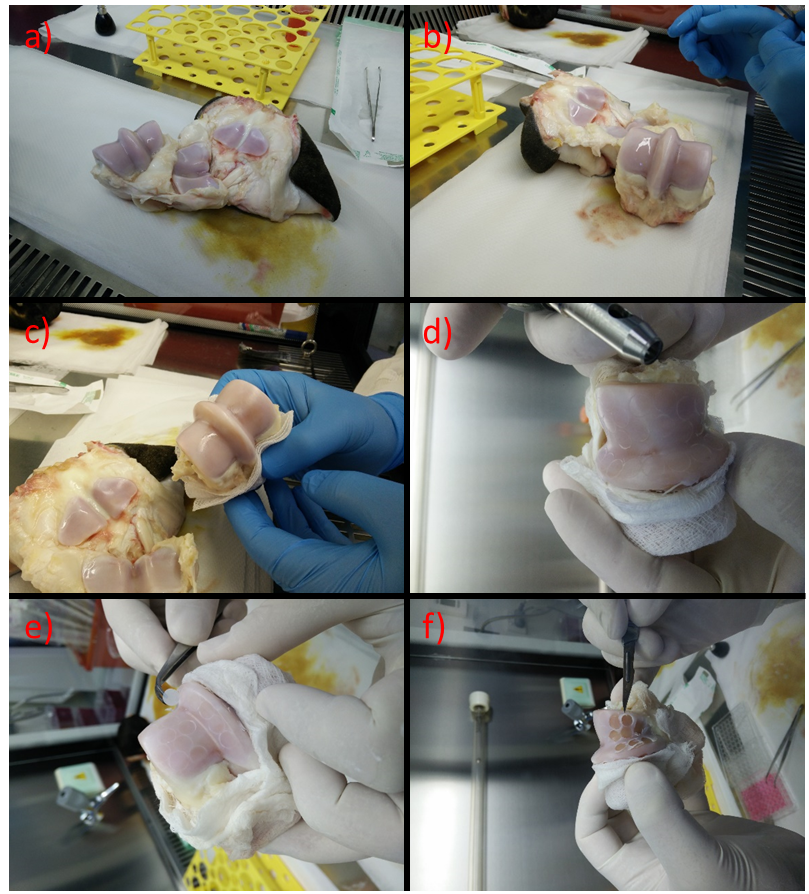 |
| --- |
| **Supplemental figure 2 (SF 2) Articular cartilage disc sectioning protocol**  Images depict articular cartilage disc sectioning protocol conducted in a class II sterile safety cabinet. **a)** the fetlock joint was initially dis-articulated and the joint capsule flared open **b)** the third metacarpal is released from the fetlock joint **c)** once the third metacarpal is completely liberated it was wrapped in sterile gauze and the articular joint face was soaked in PBS for 5 minutes **d)** the articular cartilage was then perforated using a custom designed hollow cylindrical instrument which measured 6.45 mm in diameter equal to a standard 96-well plate diameter **e)** perforated discs were sliced off **f)** discs are stored in the correct orientation (joint surface up) in a 96-well plate containing DMEM and frozen for later use. |

|  |
| --- |
| **Supplemental figure 3 (SF 3) falling-ball test to measure viscosity**  Falling-ball viscometry was used to determine the viscosity of HA media (0.5-5 mg/ml) relative to control media. A 5 ml serological pipette with a diameter of 5.9 mm was filled with media pre-warmed to 37°C. A bio-silicate sphere, 3 mm in diameter was dropped and time measured between two defined points with a known distance. A total of five replicates for each media were obtained. The data was expressed as a mean flow rate (millimetres per second) and the stand deviation as error bars. |

| 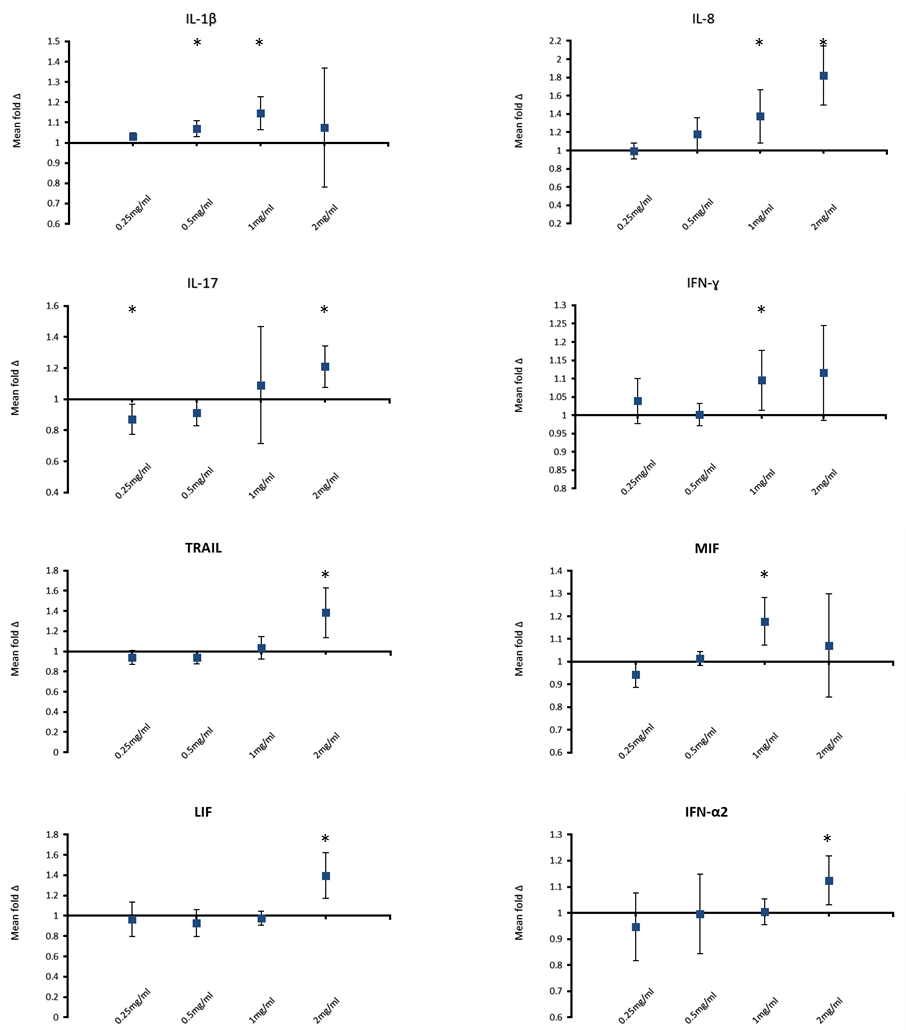 |
| --- |
| **Supplemental figure 4 (SF 4) fold change of pro-inflammatory cytokine secretion with HA treatment**  Conditions were all cultured in either control media or HA media for three days after the media change. Conditions *(x-axis)* were grown in flasks (B-E) HA media, ranging from 0.25 to 2 mg/mL of HA. These were all compared back to the control condition (cells grown control media, flask (A)). Average fold change in fluorescence *(n=3)* ± upper & lower confidence intervals at 95% *(y-axis)*. Cytokines with a fold change less than one indicate a decrease with HA media treatment, a fold change greater than one indicates an increase in the cytokine with HA treatment (* cytokines with a fold change clear of the axis at 1 reported as significant). Interleukin-1β (IL-1β), Interleukin-8 (IL-8), Interleukin-17 (IL-17), Interferon-gamma (IFN-ɣ), TNF-related apoptosis inducing ligand (TRAIL), Macrophage migration inhibitory factor (MIF), Leukemia inhibitory factor (LIF) and Interferon alpha-2 (IFN-α2). |

| 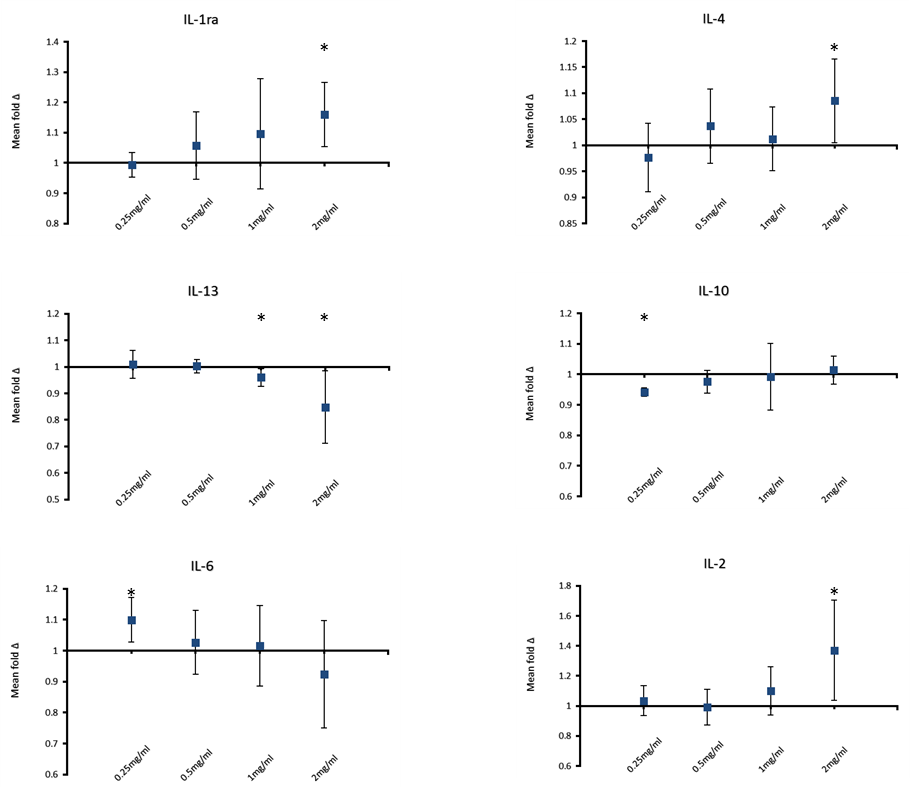 |
| --- |
| **Supplemental figure 5 (SF 5) fold change of anti-inflammatory and dual role cytokine secretion with HA treatment**  Conditions were all cultured in either control media or HA media for three days after the media change. Conditions *(x-axis)* were grown in flasks (B-E) HA media, ranging from 0.25 to 2 mg/mL of HA. These were all compared back to the control condition (cells grown control media, flask (A)). Average fold change in fluorescence *(n=3)* ± upper & lower confidence intervals at 95% *(y-axis)*. Cytokines with a fold change less than one indicate a decrease with HA media treatment, a fold change greater than one indicates an increase in the cytokine with HA treatment (* cytokines with a fold change clear of the axis at 1 reported as significant). Interleukin-1 receptor antagonist (IL-1ra), Interleukin-4 (IL-4), Interleukin 13 (IL-13), Interleukin-10 (IL-10), Interleukin-6 (IL-6) and Interleukin-2 (IL-2). |

| 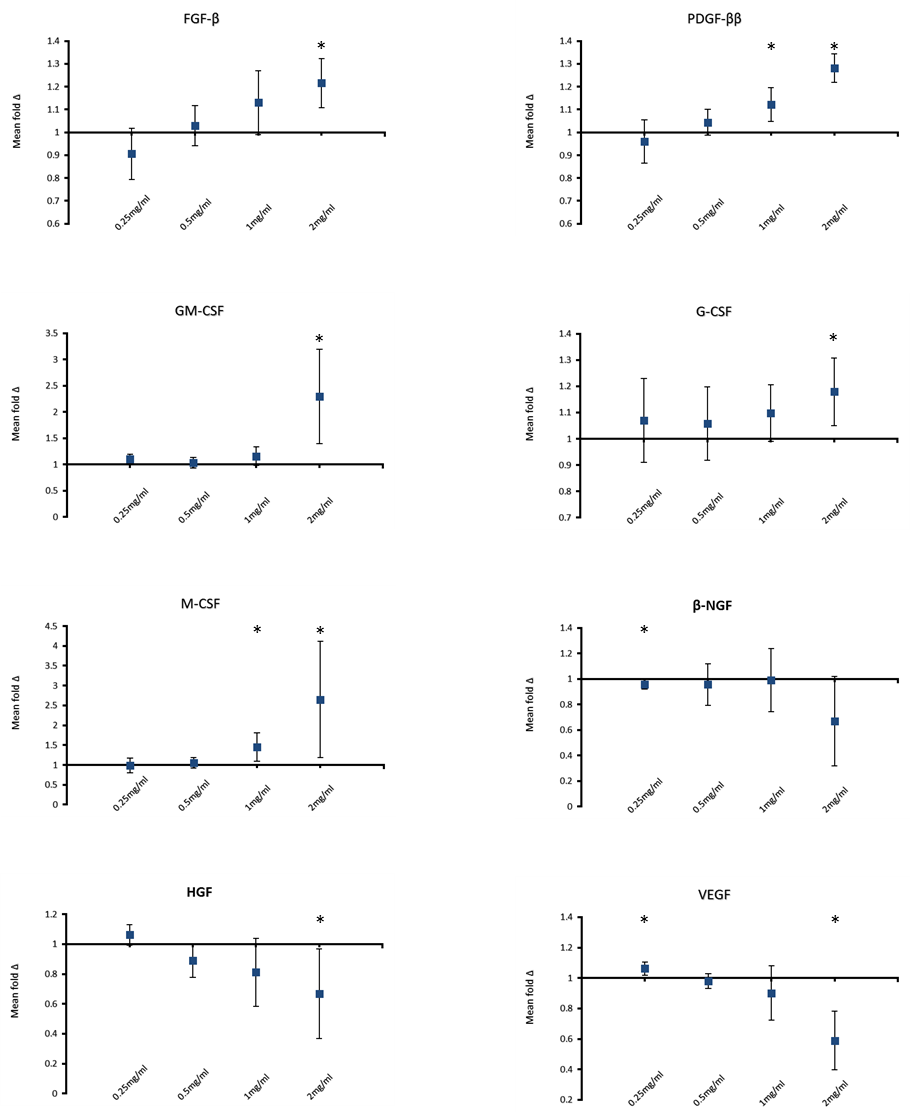 |
| --- |
| **Supplemental figure 6 (SF 6) fold change of growth factor secretion with HA treatment**  Conditions were all cultured in either control media or HA media for three days after the media change. Conditions *(x-axis)* were grown in flasks (B-E) HA media, ranging from 0.25 to 2 mg/mL of HA. These were all compared back to the control condition (cells grown control media, flask (A)). Average fold change in fluorescence *(n=3)* ± upper & lower confidence intervals at 95% *(y-axis)*. Cytokines with a fold change less than one indicate a decrease with HA media treatment, a fold change greater than one indicates an increase in the cytokine with HA treatment (* cytokines with a fold change clear of the axis at 1 reported as significant). Fibroblast growth factor-basic (FGF-β), Platelet-derived growth factor-ββ (PDGF-ββ), Granulocyte macrophage colony-stimulating factor (GM-CSF), Granulocyte colony-stimulating factor (G-CSF), Macrophage colony-stimulating factor (M-CSF), Nerve growth factor-beta (β-NGF), Hepatocyte growth factor (HGF) and Vascular endothelial growth factor (VEGF). |

| 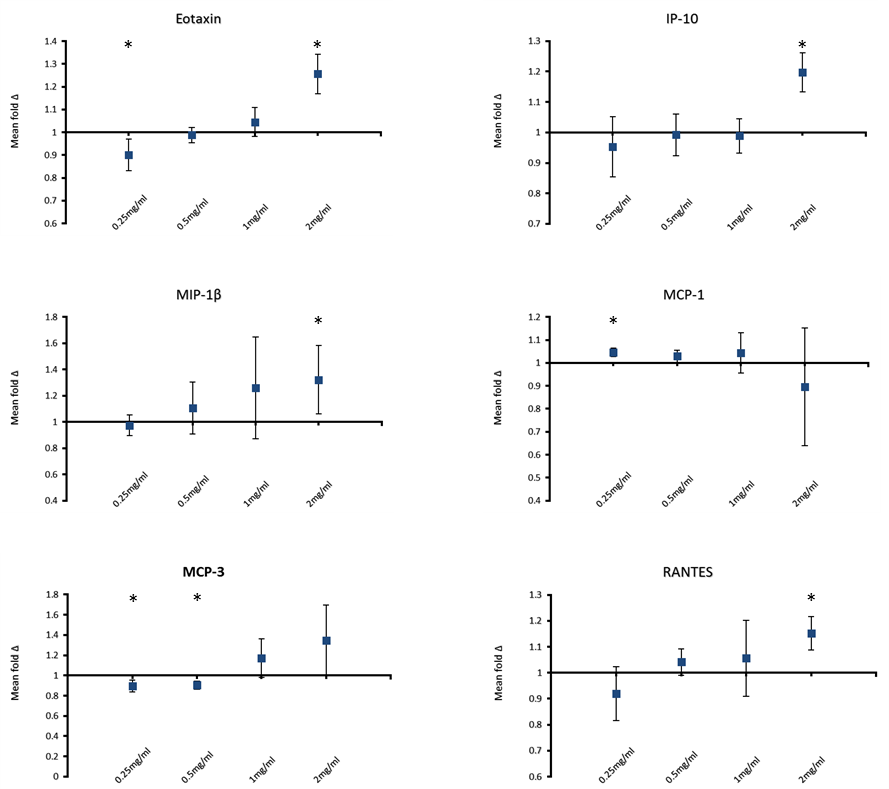 |
| --- |
| **Supplemental figure 7 (SF 7) fold change of chemokine secretion with HA treatment**  Conditions were all cultured in either control media or HA media for three days after the media change. Conditions *(x-axis)* were grown in flasks (B-E) HA media, ranging from 0.25 to 2 mg/mL of HA. These were all compared back to the control condition (cells grown control media, flask (A)). Average fold change in fluorescence *(n=3)* ± upper & lower confidence intervals at 95% *(y-axis)*. Cytokines with a fold change less than one indicate a decrease with HA media treatment, a fold change greater than one indicates an increase in the cytokine with HA treatment (* cytokines with a fold change clear of the axis at 1 reported as significant). C-C motif chemokine 11 (CCL-11) aka eosinophil chemotactic protein (Eotaxin), C-X-C motif chemokine 10 (CXCL-10) aka interferon gamma-induced protein 10 (IP-10), CC-Chemokine ligand-4 (CCL-4) aka Macrophage inflammatory protein-1 beta (MIP-1β), CC-Chemokine ligand-2 (CCL-2) aka Monocyte chemotactic protein-1 (MCP-1), CC-Chemokine ligand-7 (CCL-7) aka Monocyte chemotactic protein-3 (MCP-3), CC-Chemokine ligand-5 (CCL-5) aka Regulated on Activation Normal T Cell Expressed and Secreted (RANTES). |

|  |  | Adherence | | Proliferation | |
| --- | --- | --- | --- | --- | --- |
|  |  | *Standard Plate* | *High-adherence*  *plate* | *Standard Plate* | *High-adherence plate* |
| MSCs grown in control & seeded in HA media | *Control* | 1368 | 4627 | 2293 | 2373 |
|  | *0.25 mg* | 2642 | 4544 | 2409 | 2767 |
|  | *0.5 mg* | 2361 | 5636 | 2763 | 2684 |
|  | *1 mg* | 2658 | 4548 | ***3117*** | 3039 |
|  | *2 mg* | 1746 | ***2431*** | 2496 | 2342 |
|  |  |  |  |  |  |
| MSCs grown in HA media & seeded in control (primed) | *Control* | 1368 | 4627 | 2293 | 2373 |
|  | *0.25 mg* | 2967 | 5092 | 2506 | 2930 |
|  | *0.5 mg* | ***3990*** | 3815 | ***2736*** | ***3384*** |
|  | *1 mg* | ***3410*** | 3668 | ***3686*** | ***3840*** |
|  | *2 mg* | 2404 | ***2681*** | ***3511*** | ***2966*** |
|  |  |  |  |  |  |
| MSCs grown in HA media & seeded HA media | *Control* | 1368 | 4627 | 2293 | 2373 |
|  | *0.25 mg* | 2952 | 4830 | 2911 | 2997 |
|  | *0.5 mg* | ***3797*** | 3526 | ***3045*** | ***3681*** |
|  | *1 mg* | ***5931*** | 2778 | ***3550*** | ***3749*** |
|  | *2 mg* | 1751 | ***2119*** | ***3488*** | 3868 |
| Supplemental table 1 (ST 1) MSC growth kinetics summary  Median cell number from all growth kinetics experiments. *Bold* numbers indicate a *p-value <* 0.05 as derived from a two-sided t-test using the control as a reference for all the conditions. | | | | | |

| **Cytokines** | | **Fold Change from control (CI = 95%)** | | | | **Role in OA** |
| --- | --- | --- | --- | --- | --- | --- |
|  |  | **0.25 mg/mL** | **0.5 mg/mL** | **1 mg/mL** | **2 mg/mL** |  |
| **Pro-inflammatory** | MIF | 0.94 | 1.01 | **1.18** | 1.07 | Induces the release of proinflammatory cytokines, such as TNF-α, IFN-γ, IL-1β, IL-6, IL-8 and MIF levels in synovial fluid correlate to OA severity |
|  | TRAIL | 0.94 | 0.94 | 1.03 | **1.38** | Increased expression in Experimental rat OA-derived cartilage and can induce chondrocyte apoptosis |
|  | LIF | 0.96 | 0.93 | 0.98 | **1.40** | Increased levels in OA-derived synovial fluid |
|  | IL-1β | 1.03 | **1.07** | **1.15** | 1.08 | Induces inflammatory reactions and catabolic effects independently as well as in combination with other mediators in OA with respect to the articular cartilage |
|  | IL-8 | 1.00 | 1.18 | **1.37** | **1.82** | Increased levels in OA-derived synovial fluid and serum |
|  | IL-17 | **0.87** | 0.91 | 1.09 | **1.21** | Inhibits the synthesis of proteoglycans in OA and upregulates catabolic enzymes which break down cartilage |
|  | IFN-ɣ | 1.04 | 1.00 | **1.10** | 1.12 | Stimulate cartilage breakdown by production of enzymes via IL-1 β |
|  | IFN-α2 | 0.95 | 1.00 | 1.01 | **1.12** | Induces immunosuppressive enzyme; indoleamine-2,3-dioxygenase (IDO) |
| **Anti-inflammatory / Dual role** | IL-1ra | 0.99 | 1.06 | 1.10 | **1.16** | Blocks interactions between cell surface receptors and IL-1 inhibiting inflammatory cascades in OA |
|  | IL-4 | 0.98 | 1.04 | 1.01 | **1.09** | Can decrease the release of prostaglandin E2 in OA-derived synoviocytes and suppress synthesis of IL-1β and TNF-α in OA tissue |
|  | IL-13 | 1.01 | 1.00 | **0.96** | **0.85** | Inhibits production of proinflammatory cytokines in immune cells and increases IL-1Ra production. Inhibits IL-1β synthesis in OA-derived synovium |
|  | IL-10 | **0.94** | 0.98 | 0.99 | 1.01 | Expressed in chondrocytes and involved in collagen and aggrecan synthesis. Inhibition of catabolic enzymes and apoptosis of chondrocytes |
|  | IL-2 | 1.04 | 0.99 | 1.10 | **1.37** | Elevated levels are found in OA synovial fluid and increased levels correlate to increase severity |
|  | IL-6 | **1.10** | 1.03 | 1.02 | 0.92 | Catabolises aggrecan via aggrecanase activity. Involved in the synthesis of tissue inhibitor of metalloproteinases known to stop cartilage breakdown |
| **Chemokines** | Eotaxin | **0.90** | 0.99 | 1.05 | **1.26** | Increased concentrations in OA-derived plasma compared to healthy controls |
|  | IP-10 | 0.95 | 0.99 | 0.99 | **1.20** | Plasma and synovial concentrations are inversely associated with knee OA severity |
|  | MIP-1β | 0.97 | 1.11 | 1.26 | **1.32** | Present in significantly higher levels in OA synovial fluid compared to normal synovial fluid |
|  | RANTES | 0.92 | 1.04 | 1.06 | **1.15** | Secreted by IL-1 stimulated osteoarthritic chondrocytes and TNFα stimulated synovial fibroblasts |
|  | MCP-1 | **1.05** | 1.03 | 1.04 | 0.90 | Constitutively expressed in osteoarthritic chondrocytes and increased secretion in IL-1 stimulated osteoarthritic chondrocytes |
|  | MCP-3 | **0.90** | **0.90** | 1.17 | 1.35 | Activates immune cells such as monocytes, T lymphocytes, basophils and eosinophils |
| **Growth Factors** | HGF | 1.06 | 0.89 | 0.81 | **0.67** | Promotes osteophyte formation via MCP-1 mediated infiltration of immune cells into OA affected joint |
|  | FGF-β | 0.91 | 1.03 | 1.13 | **1.22** | Potentiates articular cartilage resurfacing and may stimulate expression of MMP-13 |
|  | β-NGF | **0.96** | 0.96 | 0.99 | 0.67 | Higher expression is found in osteoarthritic derived chondrocyte compared to healthy chondrocytes |
|  | GM-CSF | 1.11 | 1.03 | 1.15 | **2.29** | Key mediator in inflammation and arthritic pain |
|  | VEGF | **1.06** | 0.98 | 0.90 | **0.59** | Expressed in osteoarthritic-derived chondrocytes and shown to increase osteo-chondral angiogenesis in OA patients |
|  | G-CSF | 1.07 | 1.06 | 1.10 | **1.18** | Can significantly increase nitrite levels in cartilage when combined with IL-1β stimulated explants |
|  | M-CSF | 0.98 | 1.05 | **1.45** | **2.65** | Increased gene expression in articular-derived chondrocytes during inflammation |
|  | PDGF-ββ | 0.96 | 1.04 | **1.12** | **1.28** | OA-derived synovial fibroblasts stimulated with PDGF-ββ decreased total MMP activity |
| **Supplemental Table 2 (ST 2) Summary of Cytokines across all conditions**  A summary of cytokines across all the concentrations of HA treatment. The data is represented as fold change in fluorescence compared to control. A value more than one indicates the HA treatment increased the secretion of that cytokine in that condition. Bold values represent a significant fold change from control determined numerically using confidence interval set at 95%. | | | | | | |
|  |  |  |  |  |  |  |
